# RESILIENCE IN FAMILY CAREGIVERS OF ASIAN OLDER ADULTS WITH DEMENTIA: AN INTEGRATIVE REVIEW

**DOI:** 10.1093/geroni/igac059.910

**Published:** 2022-12-20

**Authors:** Thitinan Duangjina, Valerie Gruss

**Affiliations:** university of illinois Chicago, Chicago, Illinois, United States; College of Nursing, University of Illinois Chicago, Chicago, Illinois, United States

## Abstract

Resilience is the ability to rebound from adversity and the amount of resilience varies depending on the sociocultural contexts. Little is known about resilience in Asian family caregivers of older adults with dementia, although Asian countries show the fastest growth of dementia. An integrative review focused on resilience in Asian family caregivers was guided by Whittemore and Knafl (2005). A systematic search of five databases (CINAHL, EMBASE, PsycINFO, MEDLINE, PubMed) was conducted and limited to English-language empirical studies published between 2016 and 2021. A constant comparison approach was used for the data analysis. A total of 15 studies conducted in nine Asian countries were selected for review. Results revealed Asian family caregivers were most commonly adult children, female, and providing home care. Resilience was impacted by multiple interrelated factors. Two influences emerged from this review including risk factors (burden, stigma, family stress, social stress) and protective factors (positive aspect of caregiving [PAC], religiosity/spirituality, social support). Filial piety, derived from religiosity/spirituality, played a role in both risk and protective factors. Asian caregivers with a deep-rooted belief in filial piety exhibited more depression than US caregivers (d=.21, p <.05), as Asian caregivers restricted their other activities and dedicated themselves to caregiving. Other Asian caregivers who balanced a belief of filial piety experienced a PAC and were resilient. Future interventional research should focus on minimizing risk factors (burden) and maximizing protective factors (PAC and religiosity/spirituality) to promote resilience among Asian family caregivers. A culturally aligned family-centered approach to care should be considered.

